# Regulation of Translation Factor *EEF1D* Gene Function by Alternative Splicing

**DOI:** 10.3390/ijms16023970

**Published:** 2015-02-12

**Authors:** Taku Kaitsuka, Masayuki Matsushita

**Affiliations:** 1 Department of Molecular Physiology, Faculty of Life Sciences, Kumamoto University, Kumamoto 860-8556, Japan; E-Mail: kaitsuka@kumamoto-u.ac.jp; 2 Department of Molecular and Cellular Physiology, Graduate School of Medicine, University of the Ryukyus, Okinawa 903-0215, Japan

**Keywords:** elongation factor, alternative splicing, heat shock, stress response

## Abstract

Alternative splicing is an exquisite mechanism that allows one coding gene to have multiple functions. The alternative splicing machinery is necessary for proper development, differentiation and stress responses in a variety of organisms, and disruption of this machinery is often implicated in human diseases. Previously, we discovered a long form of eukaryotic elongation factor 1Bδ (eEF1Bδ; this long-form eEF1Bδ results from alternative splicing of *EEF1D* transcripts and regulates the cellular stress response by transcriptional activation, not translational enhancement, of heat-shock responsive genes. In this review, we discuss the molecular function of *EEF1D* alternative splicing products and the estimated implication of human diseases.

## 1. Introduction

In mammals, individual coding genes can generate diverse RNA variants because of alternative splicing. Alternative splicing and the resulting RNA variants can have crucial roles in development, differentiation and stress responses [1,2]. The mammalian brain expresses numerous RNA variants from individual genes [3] via alternative splicing; moreover, related RNA variants from a single gene can participate in neuronal development and neurodegenerative diseases [4]. Previously, we discovered that *EEF1D*, a gene encoding eukaryotic elongation factor 1Bδ (eEF1Bδ), gives rise to a long-form *EEF1D* transcript variant, specifically in brain and testis, because of alternative splicing [5]. Furthermore, long-form *EEF1D* transcripts give rise to a translation product (designated eEF1BδL) that functions as a transcription factor; therefore, eEF1BδL function (transcriptional activation) differs critically from eEF1Bδ function (translational elongation).

## 2. *EEF1D* Gene Structure and *EEF1D* Homologs

**Figure 1:**
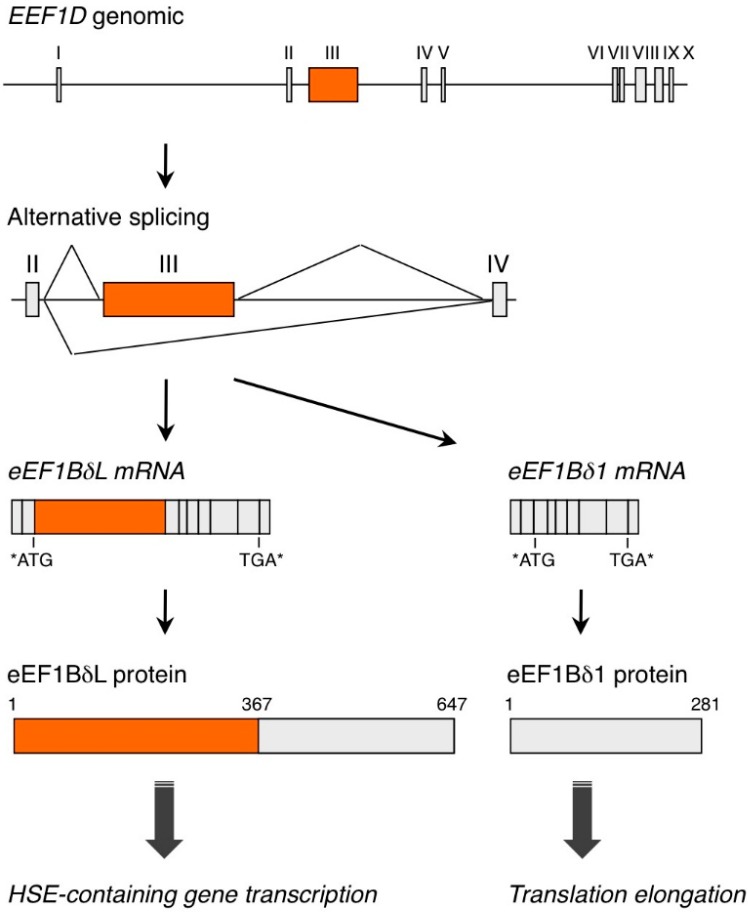
Schematic illustration of the *EEF1D* gene and protein products. Short- or long-isoform eEF1Bδ mRNA is expressed, depending on whether exon III is skipped. eEF1Bδ functions as a guanine nucleotide exchange factor for eEF1A and has a crucial role in translation fidelity; eEF1BδL functions as a transcription factor for HSE-containing genes. The numbers of amino acids are shown. Asterisks indicate start or stop codons. eEF1Bδ, eukaryotic elongation factor 1Bδ; eEF1BδL, long isoform of eEF1Bδ; HSE, heat-shock element; mRNA, messenger RNA.

**Figure 2:**
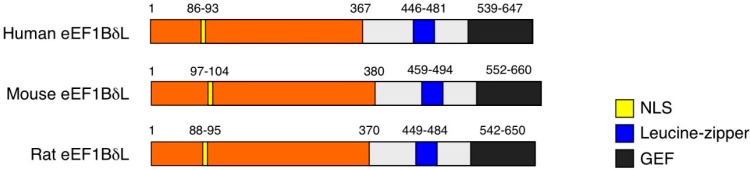
Comparison of eEF1BδL structural domains between human and rodent orthologs. The nuclear localization signal (NLS), leucine-zipper motif and guanine nucleotide exchange factor (GEF) domain are well conserved between human and rodent orthologs. The numbers of amino acids are shown.

The *EEF1D* gene is located on chromosome 8 in humans and on chromosome 15 in mice. In humans, the *EEF1D* gene gives rise to four protein isoforms, which are designated isoform 1, 2, 4 or 5 in gene-centered information at NCBI (Gene ID 1936). These isoforms can be divided into two types based on polypeptide length. One type includes only one long isoform with 647 aa, which is designated isoform 1 in protein-centered information at NCBI (Accession Number NP_115754), named eEF1BδL (Figure 1) [5]. Another type includes three short isoforms, each called eEF1Bδ and comprising 281, 257 or 262 aa; these proteins are designated isoform 2, 4 or 5, respectively, in protein-centered information at NCBI (Accession Number NP_001951, NP_001123528, NP_001182132, respectively) [6]. The canonical eEF1Bδ protein was initially isolated from *Xenopus oocytes* [7] and secondarily characterized in humans [8]. This eEF1Bδ protein mainly localizes in cytoplasm and acts as a translation elongation factor. This protein has a leucine-zipper motif (Figure 2) and forms a complex with eEF1Bα, eEF1Bγ and valine-tRNA synthetase; this complex catalyzes the exchange of guanosine 5'-diphosphate, which binds to G-protein eEF1A in the elongation cycle. Thus, eEF1Bδ functions as a guanine nucleotide exchange factor (GEF) for eEF1A [6,9,10]. By contrast, eEF1BδL localizes in cytoplasm and nuclei under basal conditions and acts as a transcription factor for genes that contain heat-shock elements (HSEs) [5]. Human eEF1BδL has 367 more aa at its *N*-terminus than does eEF1Bδ, and this *N*-terminal eEF1BδL sequence contains a nuclear localization signal (NLS) (Figure 2). Furthermore, we found that: (1) eEF1BδL induces *HSPA6*, *CRYAB*, *DNAJB1* and *HMOX1* transcription in cooperation with heat-shock transcription factor 1 (HSF1) and NF-E2-related factor 2 (Nrf2); (2) eEF1BδL binds directly to HSE oligo DNA *in vitro* and associates with the HSE consensus in the *HMOX1* promoter region *in vivo*; (3) heat shock induces the splicing-dependent change from eEF1Bδ to eEF1BδL expression; and (4) translocation of eEF1BδL into the nucleus is facilitated by treatment with the protease inhibitor, MG132.

**Figure 3:**
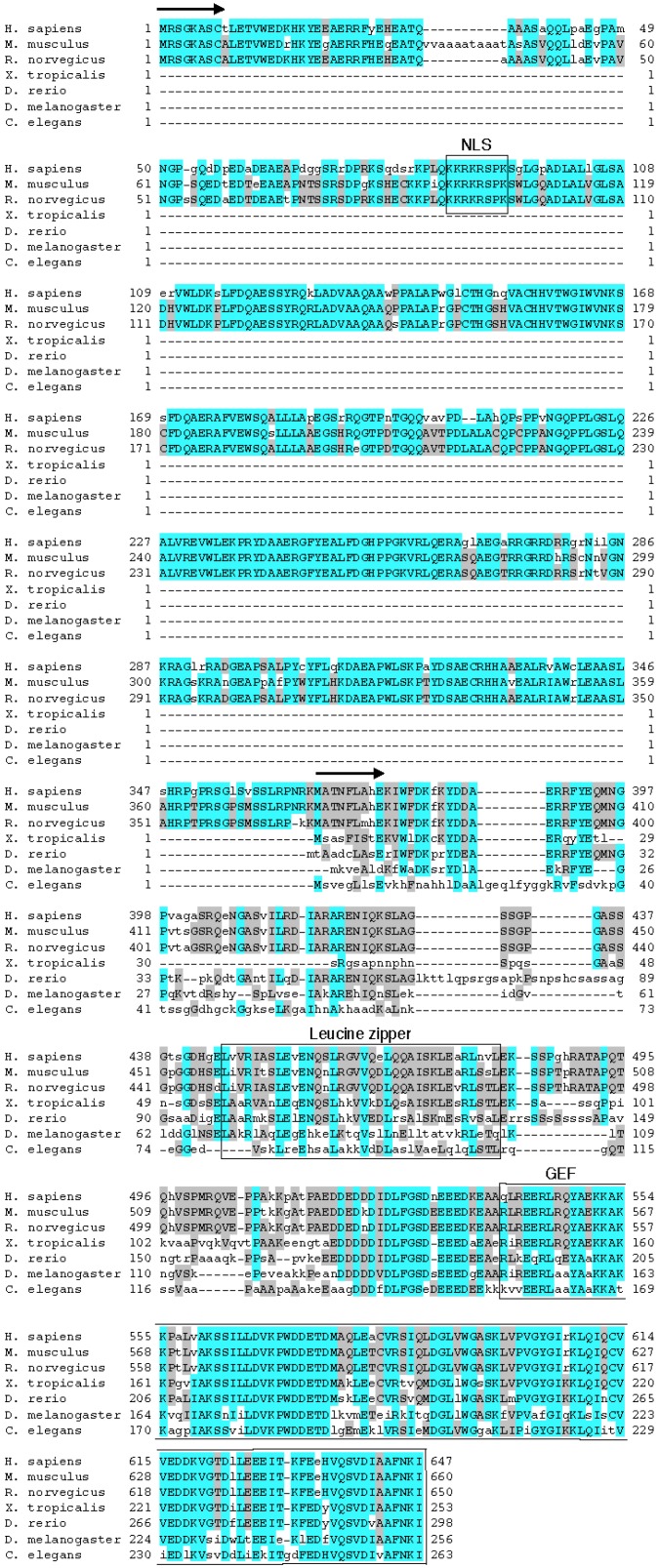
Alignment of the amino acid sequences of mammalian eEF1BδL and other eukaryotic eEF1Bδ proteins. Within the compared sequences, the blue highlights show primary conserved regions, and the gray highlights show secondary conserved regions. Arrows indicate each *N*-terminus of the mammalian eEF1BδL and other eukaryotic eEF1Bδ proteins. The nuclear localization signal (NLS), leucine-zipper motif and guanine nucleotide exchange factor (GEF) domain are marked by boxes and text. MUSCLE was used to create the alignment [12].

Canonical eEF1Bδ is expressed in almost all metazoan species tested, including worm and human, and these metazoan eEF1Bδ orthologs are highly homologous by 65% (gi|25453472 *vs.* gi|71997105). In contrast, the expression of eEF1BL is restricted to mammals and avians [11] (Figure 3). *Xenopus tropicalis*, *Danio rerio*, *Drosophila melanogaster* and *Caenorhabditis elegans* do not express eEF1BδL (Figure 3). The *C*-terminal region of eEF1BδL, *i.e.*, canonical eEF1Bδ, is highly conserved from worm to human, especially the leucine-zipper motif and GEF domain. The nuclear localization signals in the *N*-terminal region of eEF1BδL orthologs are highly conserved from rat to human (Figure 3). Thus, eEF1BδL may have emerged in the lineage leading from reptiles to avians and mammals. Two interesting structural features are apparent in the *N*-terminal region. First, the highly-conserved leucine-rich zipper-like region at aa 184–225 of human eEF1BδL suggests a protein interaction domain. Second, the basic region at aa 272–294 suggests a DNA binding domain, although the functional significance of both regions is unclear at present.

Furthermore, *eEF1BδL* RNA and eEF1BδL protein are enriched in brain and testis [5]. Alternative splicing has important roles in the control of neuronal gene expression, neuronal development and synaptic strength [13,14]. For instance, the *Dscam* gene regulates the formation of the neuronal circuit by alternative splicing machinery [15], and neurexin splice variants are involved in synapse formation [16]. Ca^2+^-channels, including the Cav2, channel are also regulated by alternative splicing in neuronal tissues [17]. Thus, *EEF1D* expression was newly discovered to be regulated by brain- and testis-specific alternative splicing.

## 3. eEF1BδL Target Genes

eEF1BδL can localize to nuclei and induce HSE-containing genes, such as *HSPA6*, *DNAJB1*, *CRYAB* and *HMOX1*, in cooperation with HSF1 and Nrf2 [5]. *HSPA6*, also known as *HSP70B’*, is a HSP70 family member. *HSPA6* is a strictly stress-inducible gene, and this expression is undetectable in most cells under non-stressed conditions [18,19]. Interestingly, orthologs of the human *HSPA6* gene are found in *Bos taurus*, but not in rodents or lower species [20]. *HSPA6* is induced by heat shock and proteasome inhibition [20,21] and contributes to cell survival under conditions of stress [20,21]. *CRYAB*, also known as *HSPB5*, is also a heat-shock-responsive gene, and the protein product has a chaperone-like activity [22]. *DNAJB1* is a major Hsp40 member that regulates Hsp70 in mammalian cytosol [23], and the *DNAJB1* protein product reportedly suppresses polyglutamine toxicity [24]. Transcription of *HMOX1* is activated by many transcription factors that regulate cellular stress responses; these transcription factors include HSF family members, Nrf2, nuclear factor-κB (NF-κB) and activator protein-1 (AP-1) [25]. The *HMOX1* protein product, HO-1, is an enzyme that catalyzes the rate-limiting reaction in heme catabolism; consequently, HO-1 has cytoprotective activity against oxidative stress [25]. Almost all eEF1BδL target genes are responsive to heat shock or some other stress, and these are cytoprotective genes to ameliorate protein aggregation and oxidative insult.

## 4. The Role of eEF1Bδ and eEF1BδL in Stress Response

The activity of eEF1BδL is modulated by some stress responses, including the unfolded-protein response. The splicing-dependent switch from eEF1Bδ to eEF1BδL expression is induced by heat shock [5]. Gene transcription and mRNA translation are responsive to various stresses. In the case of mRNA translation, repression of the translation machinery often occurs as an adaptation to a number of stresses, such as thermal stress or hypoxic stress; therefore, mRNA translation factors are important in stress responses and human diseases [26,27,28,29]. Such stresses trigger the phosphorylation of eukaryotic initiation factor eIF2α at Ser51 [26,28,30,31]. This inhibits the exchange of GDP for GTP on the eIF2 complex and prevents the formation of the eIF2-GTP-Met-tRNA_i_^Met^ ternary complex [30,31]. Hypoxic stress and energy starvation also activate AMP-activated kinase (AMPK), and activated AMPK phosphorylates eEF2 kinase (eEF2K) at Ser398 and activates its kinase activity [32,33]. eEF2K then phosphorylates eEF2 at Thr56, resulting in the inhibition of peptide elongation [34]. Repression of translation results in a substantial saving of cellular energy, which is mainly consumed in the process of translation [26,35,36,37]; this prevents the synthesis of unwanted proteins and, therefore, protects cells by reducing the toxicity caused by unfolded proteins [29]. Loss or inactivation of eEF1B, eIF4E or aminoacyl-transfer RNA synthetases, which each regulate mRNA translation, enhances a cell’s resistance to stress [27,29,38]. Additionally, various stresses can elicit the activation of specific transcription factors, which then induce stress-response genes and rescue a cell; such transcription factors include HSF1 [39], Nrf2 [40] and hypoxia-inducible factor (HIF) [41]. In our work, heat shock reduced eEF1Bδ expression, but it simultaneously increased eEF1BδL expression in a splicing-dependent manner. Taken together, these finding indicate that one coding gene, *EEF1D*, would participate in regulating two stress-response mechanisms: generalized translational repression and transcriptional activation of stress-response genes. Furthermore, eEF1BδL activity is also regulated via translocation from the cytoplasm to the nucleus by MG132, causing the accumulation of unfolded proteins [5].

eEF1BδL contains a canonical eEF1Bδ sequence at its *C*-terminal region, and this region is necessary to support the transcriptional activity of its protein [5]. To examine whether canonical eEF1Bδ has the potential to activate the transcription of the HSE-containing genes, we constructed a plasmid vector expressing eEF1Bδ fused to NLS. Overexpression of NLS-eEF1Bδ did not induce *HMOX1* expression in HEK293 cells (unpublished data), indicating that the *N*-terminal region of eEF1BδL is also essential for the transcriptional activity of eEF1BδL. Canonical eEF1Bδ has a leucine zipper motif and forms a macromolecular complex with eEF1Bα, eEF1Bγ and valine-tRNA synthetase [6]. Therefore, it is possible that eEF1BδL also forms a complex with these subunits of elongation factors or with other proteins in the nucleus. While it is unclear whether eEF1BδL has GEF activity for eEF1A and participates in mRNA translation, further study is needed to clarify this question.

## 5. A Putative Role for eEF1BδL *in Vivo*

Most HSE-containing genes, especially those encoding heat shock proteins (Hsps), code for molecular chaperones that were originally defined because of an HSF1-dependent increase in expression in response to cellular stressors, such as thermal and oxidative stress [39,42]. Molecular chaperones have essential roles in protein homeostasis, prevent misfolding and aggregation of proteins and allow the clearance of damaged proteins [43,44]. Members of the HSF family are the transcription factors mainly responsible for Hsps induction. In response to various inducers, such as heat shock, most HSFs acquire DNA binding activity and bind to HSEs, thereby inducing the transcription of genes encoding Hsps [39,45,46,47]. Among the family of HSFs (*i.e*., murine and human HSF1, 2 and 4), HSF1 is an HSF prototype and a prime integrator of transcriptional responses during stress [47,48]. *Hsf1*-knockout mice and cell models reveal that HSF1 is a prerequisite for the transactivation of Hsp genes, the maintenance of cellular integrity during stress and the development of thermotolerance [49]. *Hsf1*-knockout mice also reveal that maternal HSF1 regulates embryo development [50], postnatal inflammatory responses [51] and carcinogenesis [52]. However, the HSF1 response to cellular stresses is absent in mature neurons in the adult brain [43]; this finding indicates that other machinery is required for Hsps expression in these adult neural tissues; such machinery may include eEF1BδL. Protein misfolding in neuronal tissues is implicated in Huntington’s disease, Parkinson’s disease, familial amyotrophic lateral sclerosis and Alzheimer’s disease [43,53]. eEF1BδL is specifically expressed in the brain, indicating the possibility that this protein could participate in the pathogenesis of these diseases. Nrf2, which is a basic leucine-zipper transcription factor, plays a crucial role in the inducible cell defense system. During chemical exposure and/or oxidative stress, Nrf2 activates the transcription of cytoprotective genes [40,54]. Oxidative stress-response pathways are implicated as a major cause of brain stroke and some other neurodegenerative diseases, such as Parkinson’s and Alzheimer’s diseases [54]. eEF1BδL and Nrf2 interact with each other in the promoter of a shared target gene, *HMOX1* [5]; these findings also indicate the possibility that eEF1BδL could correlate with brain stroke and neurodegenerative diseases. Furthermore, rare variants in *EEF1D* gene were found in late-onset familial Parkinson’s diseases [55], supporting a possible correlation between *EEF1D* and the pathogenesis of this disease. In the case of testis, Hsfs, especially Hsf2, is also essential for spermatogenesis and male fertility [56,57,58]. Notably, the strong expression of eEF1BδL in human testis indicates the possibility that eEF1BδL has a role in male fertility.

## 6. Conclusions

As discussed, the expression of eEF1BδL might be taxonomically restricted to avians and mammals, suggesting that the regulation of *EEF1D* gene expression by alternative splicing is an avian- and mammalian-specific phenomenon. The orthologs of eEF1BδL are not found in reptiles or lower species. Furthermore, higher expression is detected in the cerebrum and cerebellum. We propose that evolution from reptiles to avians and mammals may have required additional proteins that could regulate protein homeostasis in the brain because of the substantial change in brain structure and function in the avian and mammalian lineage. Further study involving knockout mice and human clinical specimens is needed to clarify the role of eEF1BδL in normal mammalian physiology and pathophysiology.
